# The Effects of GLP-1 Receptor Agonists on Retinal Microvascular Alterations

**DOI:** 10.3390/biomedicines14051057

**Published:** 2026-05-07

**Authors:** Stamatios Lampsas, Gerasimia-Marina Chardalia, Chrysa Agapitou, Konstantinos Papastamopoulos, Panagiotis Theodossiadis, Gerasimos Siasos, Evangelos Oikonomou, Vaia Lambadiari, Irini Chatziralli

**Affiliations:** 1 Second Department of Ophthalmology, School of Medicine, National and Kapodistrian University of Athens, “Attikon” University Hospital, 12462 Athens, Greece; gerasimiamarina@gmail.com (G.-M.C.); chr.agapitou@gmail.com (C.A.); papastamk@gmail.com (K.P.); patheo@med.uoa.gr (P.T.); eirchat@med.uoa.gr (I.C.); 2 Third Department of Cardiology, School of Medicine, National and Kapodistrian University of Athens, “Sotiria” General Hospital, 11527 Athens, Greece; ger_sias@hotmail.com; 3 Cardiovascular Division, Harvard Medical School, Brigham and Women’s Hospital, Boston, MA 02115, USA; boikono@gmail.com; 4 Second Department of Internal Medicine, Research Institute and Diabetes Center, School of Medicine, National and Kapodistrian University of Athens, “Attikon” University Hospital, 12462 Athens, Greece; vlambadiari@gmail.com

**Keywords:** GLP-1 receptor agonists, diabetic retinopathy progression, retinal microvascular dysfunction, ocular adverse events, endothelial dysfunction, inflammation

## Abstract

Glucagon-like peptide-1 receptor agonists (GLP-1RAs) have revolutionized the management of type 2 diabetes mellitus (T2DM) by providing robust glycemic control alongside significant cardioprotective and renoprotective benefits. This review synthesizes current mechanistic, preclinical, and clinical evidence regarding the impact of GLP-1RAs on retinal microvasculature and summarizes the current clinical evidence of GLP-1RA-induced retinal complications. GLP-1RAs exert pleiotropic effects on the retinal microvasculature, offering protection by amelioration of endothelial function, reduction in oxidative stress, inflammation, microvascular remodeling, and preservation of the blood–retinal barrier (BRB). Despite these mechanistic advantages, emerging clinical data have raised concerns regarding potential retinal adverse events associated with GLP-1RA therapy. Observational studies and pharmacovigilance analyses have suggested possible associations with non-arteritic anterior ischemic optic neuropathy (NAION), diabetic macular edema (DME), vitreous hemorrhage, retinal detachment, macular hole formation, and progression of diabetic retinopathy (DR), particularly in the context of semaglutide use. Most evidence comes from retrospective studies or secondary endpoints, limiting causal inference. Retinal complications associated with GLP-1RAs remain heterogeneous and inconclusive, requiring careful evaluation of potential risks across diverse patient populations. Future research should conduct large, randomized trials with standardized ocular endpoints, detailed imaging, and stratified analyses to clarify GLP-1RA retinal safety.

## 1. Introduction

Glucagon-like peptide-1 receptor agonists (GLP-1RAs) have fundamentally altered the therapeutic landscape of type 2 diabetes mellitus (T2DM) treatment by coupling robust glycemic modulation with clinically significant cardioprotective and renoprotective benefits [1,2]. Pivotal trials, like SUSTAIN-6, LEADER, REWIND, and PIONEER-6, have proven the cardioprotective effect of GLP-1RAs, as well as their role in better glycemic control and weight loss, with a low risk of hypoglycemia. However, GLP-1RAs have also been found to have pleiotropic metabolic actions on endothelial function, oxidative stress, inflammation, and vascular remodeling—pathways central to the pathogenesis of diabetic microvascular complications [3,4,5,6].

Retinal microvasculature consists of a complex and highly specialized vascular network, including arterioles, capillaries, and venules, which are arranged into distinct superficial and deep capillary plexuses that ensure the accurate delivery of oxygen and essential nutrients to the metabolically demanding neural tissue [7,8]. Retinal microvascular structural and functional integrity is orchestrated by tightly regulated interactions among endothelial cells, pericytes, glial cells, and components of the extracellular matrix, which together preserve the inner blood–retinal barrier (BRB) and strictly regulate vascular permeability as well as blood flow autoregulation [9,10,11]. Endothelial dysfunction is a precursor point in the development of microangiopathy in diabetic patients driven by hyperglycemia-induced oxidative stress, advanced glycation end products, NF-κB activation, and reduced nitric oxide bioavailability, disrupting the endothelial glycocalyx and blood–retinal barrier and promoting vascular permeability, inflammation, and capillary nonperfusion, leading to diabetic retinal complications [12,13].

GLP-1RAs exert multifaceted protective effects on the retinal microvasculature. Particularly, they enhance nitric oxide (NO) bioavailability through activation of several metabolic pathways, promoting endothelial nitric oxide synthase (eNOS) phosphorylation and restoring endothelial-dependent vasodilation [14]. Moreover, GLP-1RAs enhance vascular barrier integrity by preserving endothelial junctions and the glycocalyx, reducing apoptosis, and suppressing adhesion molecules and matrix metalloproteinases, thereby limiting leukocyte adhesion, vascular remodeling, and microvascular hyperpermeability, including at the blood–retinal barrier [15]. Furthermore, GLP-1RAs attenuate microvascular inflammation by reducing oxidative stress, and mitochondrial reactive oxygen species (ROS) production, leading to decreased expression of pro-inflammatory cytokines and adhesion molecules [16]. Activation of GLP-1 receptors within the retina confers protective effects on endothelial cells, pericytes, retinal ganglion cells, and retinal pigment epithelial cells by reducing apoptosis, oxidative injury, and inflammatory signaling [17].

Although GLP-1RAs exhibit well-established microvascular protective effects, emerging evidence has raised concerns regarding their potential association with retinal adverse events, including non-arteritic anterior ischemic optic neuropathy (NAION), diabetic macular edema (DME), macular hole, retinal detachment, vitreous hemorrhage and diabetic retinopathy (DR) progression. Given these conflicting data and the widespread and increasing use of GLP-1RAs, this literature review aims to shed light on the current evidence of GLP-1RA-induced retinal complications.

## 2. Materials and Methods

This narrative review was conducted based on a non-systematic but structured search of the literature using PubMed and Scopus databases. While no predefined restrictions were applied, greater emphasis was placed on studies published within the last decade to ensure the inclusion of more recent evidence. Furthermore, relevant clinical guidelines and trial registries were examined, where applicable, to ensure the inclusion of current clinical insights and safety data regarding GLP-1RAs and their effects on retinal outcomes. Search terms included combinations of “GLP-1 receptor agonists”, “semaglutide”, “liraglutide”, “dulaglutide”, “exenatide”, “tirzepatide”, “diabetic retinopathy”, “diabetic macular edema”, “retinal microvasculature”, “endothelial dysfunction”, “blood–retinal barrier”, “oxidative stress”, “inflammation”, “NAION”, “vitreous hemorrhage”, “retinal detachment” and “macular hole”. Eligible studies included experimental and observational studies, randomized trials, pharmacovigilance analyses, meta-analyses, and relevant clinical guidance addressing GLP-1RAs and retinal microvascular or ocular outcomes. Non-peer-reviewed articles, irrelevant studies, and publications with insufficient methodological detail were excluded. Evidence was prioritized according to study design, with randomized trials, meta-analyses, and large cohort studies considered higher-level evidence. Preclinical studies were included when they provided mechanistic insight. Given the narrative design, no formal systematic review protocol or quantitative synthesis was performed.

## 3. GLP-1RAs and Microvasculature

### 3.1. GLP-1RAs and Endothelial Dysfunction

The endothelium is a functionally diverse and biologically active organ that, through autocrine, paracrine, and endocrine signaling, orchestrates vascular homeostasis by regulating vessel tone, blood flow, coagulation, inflammation, and angiogenesis [18]. Retinal vascular endothelial cells form a monolayer lining the lumen of retinal blood vessels, functioning as a selective interface between the neural retina and the systemic circulation, facilitating the delivery of oxygen and nutrients to retinal tissue [19]. GLP-1RAs are involved in multiple signaling pathways that contribute to the regulation of endothelial function [20].

Clinical evidence indicates that the GLP-1RA exenatide improves vascular endothelial dysfunction in newly diagnosed patients with T2DM, with efficacy comparable to metformin, as demonstrated by improvements in digital reactive hyperemia index [21]. Respectively, another study indicates that GLP-1RAs show greater improvement in endothelial glycocalyx than insulin and sodium–glucose cotransporter-2 inhibitors (SGLT-2i) after 12-month treatment [22]. Endothelial glycocalyx serves a catalytic role in vascular homeostasis, as a gel-like layer coating the luminal surface of blood vessels that protects the vessel wall from inflammatory cell adhesion and transduces shear stress to stimulate endothelial nitric oxide (NO) release [23]. Moreover, in overweight individuals with T2DM, GLP-1RAs have been shown to enhance peripheral microvascular blood flow, as evaluated by fingertip hyperemia, and to increase capillary density as assessed using capillary videomicroscopy [24]. Preclinical and clinical studies consistently show that GLP-1RAs therapy increases endothelial nitric oxide production and promotes Endothelial Nitric Oxide Synthase (eNOS) phosphorylation via AMPK/Akt signaling [25]. Noteworthy is the fact that GLP-1RAs endothelial vascular benefits are independent of glycemic lowering in patients with many comorbidities such as hypertension, obesity, and heart failure [26]. Nitric oxide (NO) NO bioavailability is a central feature of endothelial dysfunction that, in patients with DM, is due to hyperglycemia-induced oxidative stress and upregulation of arginase activity, which competes with eNOS for the substrate L-arginine and further increases reactive oxygen species (ROS) formation [14]. Not only in vitro studies but also in vivo studies revealed increased NO bioavailability and dependent upregulation of eNOS via a GLP-1RA/cAMP/protein kinase A axis, showing protection against endothelial cell dysfunction in diabetic models after GLP-1RAs administration [27].

### 3.2. GLP-1RAs and Microvascular Permeability

Disruption of microvascular fluid permeability is a common and often severe complication in critically ill individuals and is also frequently observed in patients with diabetes, particularly in the presence of microvascular complications such as retinopathy and nephropathy [28]. Findings from an in vivo rat model suggest that GLP-1RAs limit inflammation-induced microvascular hyperpermeability by exerting direct endothelial protective effects through receptor-mediated signaling that preserves intercellular junction integrity and inhibits apoptosis, a mechanism supported by its known antiapoptotic actions during endothelial ischemic and inflammatory injury [29]. Moreover, in T2DM models, GLP-1RAs enhance endothelial function by downregulating adhesion molecules and matrix metalloproteinases, thereby reinforcing vascular barrier integrity and limiting leukocyte passage across the endothelium, which reverses vascular remodeling and further stabilizes the vascular barrier [15]. Furthermore, the blood–retinal barrier is further supported by GLP-1RAs-induced decreased oxidative stress and increased nitric oxide availability, which together help maintain endothelial integrity and reduce vascular permeability under normoglycemic conditions [30].

### 3.3. GLP-1RAs and Oxidative Stress/Microvascular Inflammation

GLP-1RAs significantly improve inflammation and oxidative stress profiles due to their strong anti-inflammatory actions at the endothelial–immune cell interface, suppressing critical mechanisms involved in leukocyte adhesion and recruitment [31]. Particularly, GLP-1RAs mediate reduction in reactive oxygen species (ROS) production, as they mitigate hyperglycemia-induced oxidative damage in endothelial cells by downregulating NADPH oxidase activity and mitochondrial superoxide generation [32]. Moreover, GLP-1RAs activation increases intracellular cAMP, leading to PKA and PI3K/Akt pathway activation, which phosphorylates eNOS and enhances nitric oxide (NO) production, thereby improving endothelial function [33]. GLP-1RAs activation inhibits Nuclear Factor kappa-light-chain-enhancer of activated B cells (NF-κB) degradation, prevents NF-κB nuclear translocation, and suppresses TNF-α-induced transcription of pro-inflammatory genes such as Vascular Cell Adhesion Molecule-1 (VCAM-1), Intercellular Adhesion Molecule-1 (ICAM-1), E-selectin, and Monocyte Chemoattractant Protein-1 (MCP-1), ultimately reducing monocyte adhesion and early atherogenesis [34,35,36]. Moreover, GLP-1RAs significantly reduce pro-inflammatory cytokines by suppressing NLRP3 inflammasome activation, leading to decreased cytokine production in various tissues—including TNF-α, Interleukin-6 (IL-6), Interleukin-1 beta (IL-1β), and C-reactive protein (CRP)—thereby reducing both circulating inflammatory burden and local endothelial inflammatory signaling in patients with T2DM [16].

**Figure 1:**
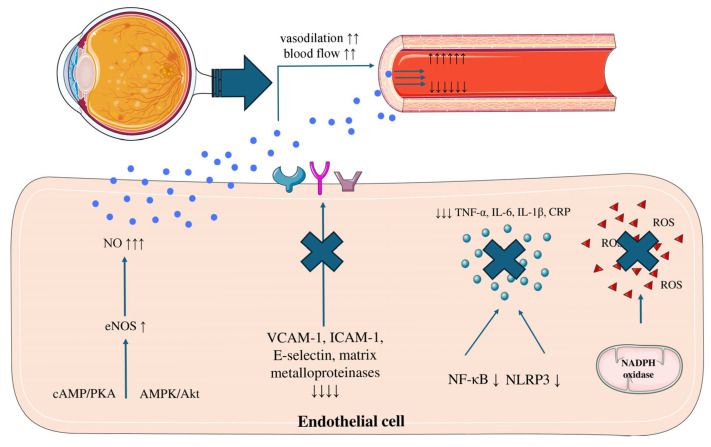
The effects of glucagon-like peptide-1 receptor agonists (GLP-1RAs) on retinal microvasculature. GLP-1RAs promotes endothelial nitric oxide synthase (eNOS) activity via activation of intracellular signaling pathways (cAMP/PKA and AMPK/Akt), resulting in elevated nitric oxide (NO) production and subsequent vasodilation with increased blood flow. Concurrently, endothelial activation markers, including vascular cell adhesion molecule-1 (VCAM-1), intercellular adhesion molecule-1 (ICAM-1), E-selectin (E-selectin), and matrix metalloproteinases (MMPs), are downregulated. Inflammatory signaling pathways are suppressed, as indicated by reduced nuclear factor kappa B (NF-κB) and NOD-like receptor pyrin domain-containing protein 3 (NLRP3) activity, leading to decreased levels of pro-inflammatory cytokines, including tumor necrosis factor-alpha (TNF-α), interleukin-6 (IL-6), interleukin-1 beta (IL-1β), and C-reactive protein (CRP). Oxidative stress is also attenuated through inhibition of nicotinamide adenine dinucleotide phosphate (NADPH) oxidase, resulting in decreased reactive oxygen species (ROS) generation. Collectively, these effects contribute to improved endothelial homeostasis and vascular protection.

Although clinical evidence remains limited, GLP-1RAs may offer neuroprotective benefits in neuroinflammatory disorders such as glaucoma. An observational retrospective study showed reduced glaucoma incidence among GLP-1RAs users, suggesting potential neuroprotective benefits that warrant further investigation [37]. Moreover, liraglutide showed protective effects in neurons from apoptosis by restoring the balance of proteins involved in mitochondrial fusion and fission and by enhancing autophagic flux [38]. Within the retina, GLP-1RAs have been demonstrated to exert protective effects on retinal ganglion cells, retinal pigment epithelial cells, and endothelial cells, attenuating inflammation and apoptosis-induced cellular damage [39]. Specifically, liraglutide and exenatide have been shown to protect RGC cells against high glucose- and H_2_O_2_-induced cytotoxicity [40]. These protective effects are mediated through enhanced eNOS phosphorylation, reduced reactive oxygen species (ROS) production, decreased caspase activation and apoptosis, and upregulation of antioxidant defenses such as superoxide dismutase (SOD) [41]. Similarly, several studies have demonstrated that activation of GLP-1RAs exerts protective effects in retinal pigment epithelial (RPE) cells, with exenatide specifically attenuating inflammatory injury and improving cell viability under TNF-α exposure and hyperglycemic conditions [17,42,43]. Finally, in vitro studies have shown that liraglutide reduces advanced glycation end product–induced retinal pericyte migration, indicating that the activation of GLP-1RAs in pericytes may help prevent their loss in diabetic retinopathy [44] (Figure 1).

## 4. GLP-1RAs and Retinal Complications

### 4.1. Non-Arteritic Anterior Ischemic Optic Neuropathy (NAION)

NAION is a form of ischemic optic neuritis, considered one of the most prevalent causes of impaired vision in elderly patients, especially those over 50 years old [45,46]. Its characteristic phenotype comprises a sudden, painless visual loss with a concurrent visual field defect, frequently accompanied by unilateral optic disk edema [47,48]. The exact pathophysiological mechanism of NAION remains to be elucidated, but the principal suggested theory rests on the hypoperfusion of the optic nerve head (ONH) leading to ischemic infarction events and, eventually, the apoptosis of the retinal ganglion cells [47,49,50]. Multiple risk factors have been implicated in its pathogenesis, such as systemic hypertension, atherosclerosis or arteriosclerosis, hyperlipidemia, diabetes, obstructive sleep apnea [51,52,53]. In addition, certain drugs have been linked to the development of NAION, most notably the phosphodiesterase type-5 inhibitors [54,55].

Hathaway et al. were the first to report, in their single-center observational study, an increased risk of NAION in diabetic patients receiving treatment with semaglutide, a GLP-1 receptor agonist (GLP-1RA), compared with diabetic patients treated with a different antidiabetic medication (HR: 4.28; *p* < 0.001) [56]. In the same study, there was also a statistically significant difference between the semaglutide and the non-semaglutide groups in the obese population (HR: 7.64; *p* < 0.001) [56]. These results raised awareness among the scientific community, leading to many more studies exploring this correlation. Supporting this increased-risk signal, a Danish cohort study followed, noting a 2.19-fold higher hazard of developing NAION in the diabetic population when receiving semaglutide once weekly over a period of up to 5 years [57]. Moreover, a disproportionality analysis showed an elevated SDR for semaglutide (ROR = 17.57; 95% CI = 13.93–21.90), highlighting that this does not constitute evidence of causation and further research needs to be conducted [58]. Remarkably, the literature largely examines diabetic patients on semaglutide alone; nonetheless, reports have noted increased NAION risk in those receiving tirzepatide as well [59]. Despite these findings, there have also been cohort studies reporting no significant associations between GLP-1RAs and NAION [60,61]. A large multinational cohort study thoroughly examined semaglutide’s association and did not find an increased risk either in diabetic-only or obesity-only patients [62]. Similarly, a meta-analysis based on randomized clinical trials (RCTs) did not identify an increased risk of ischemic optic neuropathies (IONs) in patients receiving GLP-1RAs [63]. These observations are consistent with the meta-analysis by Özbek et al. [64]. Conversely, Natividade et al. reported higher odds of NAION with semaglutide (OR, 3.92; 95% CI, 1.02–15.02). However, these results warrant caution given the wide confidence interval and the rarity of NAION in the studied population [65] (Table 1).

### 4.2. Diabetic Macular Edema (DME)

DME is defined as thickening of the central macular area, most commonly presented in patients with type 2 DM. The proposed mechanism involves hyperglycemia as the driver of inner blood–retina disruption, leading to the accumulation of exudative fluid in the retina [69,70]. The vascular endothelial growth factor (VEGF) plays a crucial role in this pathway, thus rendering anti-VEGF therapy the cornerstone of DME management [71].

The potential association between GLP-1RAs and DME has been investigated, as this complication of DM constitutes a major cause of blindness among diabetic patients. A few cohort studies suggest that the use of GLP-1RAs reduces the risk of incident DME, consistent with a possible protective effect of these agents [72,73]. In line with more neutral interpretation, additional studies did not demonstrate a statistically significant association [74,75,76]. Conversely, a large population-based study by Lakhani et al. [77], using the World Health Organization’s (WHO) Vigibase data, found that semaglutide, compared with metformin, was significantly linked to DME (ROR 3.87; 95% CI, 1.89–7.92). Furthermore, Wai et al. found consistently elevated DME risk with GLP-1RAs versus SGLT2 inhibitors across every time point assessed from 3 months to 3 years (RR ranging from 1.16 to 1.29) [78]. Although DME has not been evaluated as a standalone endpoint of microvascular complications with GLP-1RA treatment in an RCT, the SUSTAIN-6 trial reported an increased risk of retinopathy complications with semaglutide, including events requiring intravitreal treatment, possibly comprising cases of DME (HR 1.76; 95% CI, 1.11–2.78) [4]. The only meta-analysis directly addressing the association between GLP-1RA therapy and DME was conducted some years ago by Avgerinos et al., reporting no significant risk when comparing either with placebo (OR 0.84; 95% CI, 0.44–1.57) or with another antidiabetic agent (OR 1.14; 95% CI, 0.34–3.84) [79]. Given these inconsistent findings, further well-designed investigations, specifically targeting the GLP-1RA–DME relationship, are required.

### 4.3. Macular Hole

Macular hole refers to a defect in the foveal retinal layers. It can be categorized as either full-thickness macular hole (FTMH), which involves the disruption of all retinal layers, or lamellar macular hole (LMH), which is distinguished from FTMH by the preservation of the photoreceptor layer on OCT [80]. The main hypothesized mechanism leading to the formation of macular holes is vitreomacular traction (VMT). Clinically, patients may report metamorphopsia or impaired central vision. Management may require vitreoretinal surgery if spontaneous closure does not occur, and symptoms persist [81].

There is limited evidence regarding an association between macular hole formation and GLP-1RA therapy. To our knowledge, macular holes have not been prespecified as an endpoint in any study investigating ocular adverse events with GLP-1RAs. Nonetheless, a pharmacovigilance disproportionality analysis identified an increased SDR for macular holes with semaglutide (ROR 20.90, 95% CI 2.65–165.01) [77]. However, as previously noted, such signals do not establish causality, and in this case the wide confidence interval (CI) underscores the necessity of cautious interpretation and further research.

### 4.4. Retinal Detachment (RD)

RD refers to separation of the neurosensory retina from the retinal pigment epithelium (RPE). There are three types of RD: rhegmatogenous, tractional and exudative. Patients may present various symptoms such as visual field defects, photopsia and floaters [82]. RD management principally includes surgical techniques such as pars plana vitrectomy, pneumatic retinopexy or scleral buckle [83]. Urgent diagnosis and treatment are imperative for the restoration of visual acuity and retinal structural integrity [84].

Although tractional retinal detachment (TRD) frequently threatens vision in diabetic patients [85], its potential association with GLP-1 therapy has not been thoroughly investigated. Joo et al. [86] in their retrospective study compared GLP-1 users with SGLT-2 inhibitor users and reported that there was no statistically significant difference in the simultaneous event of vitreous hemorrhage and RD (OR 1.50, 95% CI 0.25–8.98). In the global pharmacovigilance study of Lakhani et al., there was an elevated SDR for RD with semaglutide in both FAERS and Vigibase databases (ROR 2.44; 95% CI 1.70–3.52 and ROR 8.60; 95% CI 4.28–17.29 respectively) [77]. However, in the only meta-analysis which assessed RD as an outcome, the use of GLP-1RAs did not increase the incidence of RD, irrespective of the comparator [79]. Aside from these findings, no additional investigations have been reported to date and definitive conclusions remain premature.

### 4.5. Vitreous Hemorrhage

Vitreous hemorrhage (VH) most commonly results from proliferative diabetic retinopathy (PDR), posterior vitreous detachment (PVD) or trauma [87]. The underlying mechanisms primarily include leakage from newly formed blood vessels during neovascularization, due to the absence of a normal endothelium barrier; disruption of pre-existing retinal blood vessels; and extension of an adjacent retinal hemorrhage. Management of VH comprises observation for spontaneous clearance, and if resorption does not occur, laser photocoagulation, anti-VEGF injections, and pars plana vitrectomy may be required [88].

The association between GLP-1RAs and VH has also been investigated, as VH is a known complication of diabetic retinopathy. Kim et al. reported a case of a diabetic patient, already diagnosed with severe non-proliferative diabetic retinopathy (NPDR), who was receiving dulaglutide 4.5 mg weekly and presented with VH. The authors noted that the causal mechanism was unclear but suggested that dulaglutide might have contributed. After lowering the dose by 1.5 mg per week, hemorrhage resorption occurred [89]. In support of this hypothesis, two disproportionality analyses have reported an elevated SDR for VH with semaglutide [77,90]. However, a recent cohort study of patients with pre-existing diabetes found a lower incidence of VH in the GLP-1RA group compared with the control group of no GLP-1RA use (HR 0.74; 95% CI 0.68–0.80; *p* < 0.001) [74]. In contrast to these findings, the SUSTAIN-6 trial reported that the occurrence of retinopathy complications (including VH events) was significantly higher in the semaglutide group (HR 1.76; 95% CI 1.11–2.78; *p* = 0.02), a difference that was potentially attributable to the rapid glucose lowering effect, which has previously been associated with worsening of retinopathy complications [4,91]. Supporting these trial data, the meta-analysis by Avgerinos et al. found a higher incidence of VH in patients treated with GLP-1RAs compared with those receiving placebo (OR 1.93; 95% CI 1.09–3.42) [79]. These observations raise the possibility of a potential direct mechanism of GLP-1RAs in the development of VH, which cannot currently be excluded [4] (Table 2).

### 4.6. Diabetic Retinopathy (DR)

DR is an important microvascular complication of diabetes mellitus (DM), potentially threatening patients’ vision [92]. Hyperglycemia, inflammation and retinal neurodegeneration are primary contributors to the development of DR. Moreover, it is roughly divided into non-proliferative (NPDR) and proliferative diabetic retinopathy (PDR) based on the absence or presence of neovascularization respectively [93,94].

The emergence of GLP-1RAs in the treatment of DM has led investigators to explore their potential effects on the retina. Preclinical studies in diabetic mouse models have provided evidence of neuroprotective and anti-inflammatory effects of exendin-4, liraglutide and lixisenatide [95,96,97]. In their cohort study, Zheng et al. examined the incidence of newly diagnosed DR in diabetic patients treated with GLP-1RAs versus those not receiving GLP-1RAs and concluded that there was a markedly lower risk in the GLP-1RA group (HR, 0.42; 95% CI, 0.29–0.61) [98]. In contrast, in a similarly designed cohort study of 185,066 diabetic patients, the GLP-1RAs group had a 7% increased risk of developing incident DR compared with the control group [74]. In addition, there is evidence that the combined use of GLP-1RAs with insulin is linked to a higher risk of DR. Furthermore, a disproportionality analysis based on the FDA Adverse Event Reporting System (FAERS) data identified signals of disproportionate reporting (SDR), particularly for semaglutide (ROR: 19.48, 95% CI: 15.20–24.96) and dulaglutide (ROR: 9.02, 95% CI: 7.11–11.44) [99]. In the SUSTAIN-6 randomized clinical trial [4], semaglutide was linked to a 76% higher risk of developing retinopathy complications, whereas the LEADER trial showed no increased hazard of DR with liraglutide (HR 1.15; 95% CI, 0.87–1.52) [100]. Given these conflicting findings, researchers have undertaken meta-analyses in order to provide more reliable evidence. Most RCT-based analyses do not demonstrate a significantly elevated risk of developing DR when receiving GLP-1RAs [101,102,103,104]. The meta-analysis by Kapoor et al. was consistent with this conclusion for all types of GLP-1RAs except for albiglutide, which was shown to have a strong association with early-stage DR (RR 2.18; 95% CI, 1.01–4.67) [105]. Moreover, in a meta-analysis restricted to semaglutide, a subgroup analysis demonstrated that there was an increased risk of DR only for older patients or patients diagnosed with diabetes for over a decade [106]. This heterogeneous evidence underscores the necessity for further research to reach a definitive conclusion on the relationship between GLP-1RA treatment and the development of DR.

### 4.7. Diabetic Retinopathy (DR) Progression

DR staging enables patient risk stratification and guides towards the most appropriate management. Based on the widely used diabetic retinopathy severity scale (DRSS) endorsed by the American Academy of Ophthalmology (AAO), DR is classified as either NPDR, subdivided into mild, moderate, and severe according to different fundus findings, or PDR, defined by the presence of neovascularization and/or vitreous/preretinal hemorrhage [107]. This disease-severity scale is routinely used in studies to assess DR progression.

GLP-1RAs are commonly administered to diabetic patients who have already been diagnosed with DR. Hence, it was therefore reasonable to consider whether these incretin-based agents influence the progression of this microvascular diabetic complication. On a preclinical level, a study by Cai et al. in diabetic animal models with DR showed that GLP-1RAs may exert beneficial effects by mitigating oxidative stress procedures leading to autophagy and apoptosis in the retina [108]. Corroborating these findings, a similarly designed study also found that semaglutide downregulates the expression of vascular endothelial growth factor (VEGF) and restores the structure of retinal layers, suggesting a multifactorial mechanism of retinal protection [109].

From a clinical standpoint, many studies, mostly retrospective cohort studies, have explored whether GLP-1RAs contribute to the progression of DR in patients with baseline DR. Wai et al. analyzed 60,649 eyes from the Vestrum Health Retina database and assessed the rate of DR progression during a 1-year follow-up period, using the DRSS, across different types of hypoglycemic treatment groups, including DPP-4 inhibitors, SGLT2 inhibitors, metformin, GLP-1RAs and a no-therapy group. The researchers concluded that the rate of progression among patients in the GLP-1RAs group from any baseline DR stage to a more advanced stage did not exceed the corresponding progression rate observed in patients receiving no hypoglycemic therapy [110]. Moreover, in a separate study by Wai et al., patients with pre-existing DR were divided into GLP-1RA users and SGLT2 inhibitor (SGLT2i) users to investigate potential differences pertaining to progression to PDR. Results showed that GLP-1RA users were at a significantly higher risk of experiencing such progression compared to the other group at 3 years follow-up (relative risk 1.28; 95% CI: 1.10–1.50) [78]. In line with these findings, a comparable cohort study by Lin et al. reported a significantly elevated risk of DR progression events in the GLP-1RA group compared with the SGLT2i group (subdistribution hazard ratio 1.50, 95% CI 1.01–2.23; *p* = 0.043). However, in their analysis, DR progression was not defined as deterioration calculated with the DRSS stages, but rather as the occurrence of any of the following complications: incident PDR, vitreous hemorrhage and tractional RD. Remarkably, they highlighted that the statistical significance of their results was mainly attributable to the increased risk of tractional RD in the GLP-1RA group [111]. Furthermore, there are reports of higher odds of incident PDR in patients with baseline NPDR, especially those with concomitant maculopathy, treated with tirzepatide [112]. Taken together, the aforementioned studies suggest a potential DR worsening effect of GLP-1Ras, which requires further investigation due to study limitations. The retrospective design, the limited number of events, and the misalignment in primary-outcome definition, diagnostic methods and follow-up procedures across studies (e.g., lack of thorough fundus exam, inadequate OCT change tracking, extraction of patient data from heterogenous databases without the possibility of physical, ophthalmic examination), all contribute to confounding, non-reliable findings.

The conflicting results raised mainly by two major RCTs, the SUSTAIN-6 trial, which reported statistically significant higher incidence of DR complications in subcutaneous semaglutide users compared to placebo, and the LEADER trial, which reported a non-significant association of DR progression in liraglutide users in comparison with placebo users, prompted scientists to question the potential impact of GLP-1RA on DR. In a meta-analysis including 13 RCTs with or without cardiovascular (CV) benefits, the authors found that GLP-1RAs in four major RCTs that demonstrated CV benefits in T2DM (SUSTAIN-6, LEADER, PIONEER-6 and REWIND) were significantly linked to a higher risk of rapidly worsening DR (OR 1.23, 95% CI 1.05–1.44). Hence, these results suggest that semaglutide (both in subcutaneous and oral form), liraglutide and dulaglutide may contribute to DR progression. The most prevalent pathophysiological mechanism appears to be the rapid and robust reduction in HbA1c observed with incretin-based agents [113]. Nevertheless, the authors note that a direct effect on the retina of these agents remains plausible, as the retinal expression of GLP-1 receptors is well-described [114]. Furthermore, another meta-analysis showed that GLP-1RA users were at a significantly higher risk of DR complications compared with oral antidiabetic drug (OAD) users (RR = 1.39, 95% CI (1.07, 1.80), *p* = 0.01) [115]. These findings enhance the early rapid worsening hypothesis, as the strong HbA1c-lowering action of GLP-1RAs is well-known and is mainly observed in patients with previously poor glycemic control, an effect less frequently seen with OAD therapy. Consistently, in a meta-regression, researchers concluded that the greater the reduction in baseline HbA1c was with GLP-1RA therapy, the higher the risk of DR progression [116]. The primary hypothesis includes hypoglycemia leading to decreased retinal blood flow, hypoxia and deterioration of DR. However, this effect, firstly described in intensively insulin-treated patients, appears transient and reversible in most cases [117]. It is important to emphasize that, to date, no RCT has been designed to directly examine the effect of GLP-1RA on DR progression as a primary endpoint. Current evidence originates from cardiovascular outcome trials (CVOTs), which captured incident DR and DR complications as adverse events, resulting in insufficient information, limited structured follow-up and possible under-ascertainment. The ongoing FOCUS trial, with results expected in 2027, is anticipated to shed light and confront this limitation by directly examining DR outcomes in semaglutide users (NCT03811561).

## 5. Conclusions

GLP-1RAs exert well-documented endothelial, anti-inflammatory, and antioxidative effects that may provide significant protective effects on the retinal microvasculature. Emerging clinical and pharmacovigilance data suggest a potential association between GLP-1RA therapy and certain retinal adverse events, including NAION, diabetic macular edema, macular hole, retinal detachment, vitreous hemorrhage, and diabetic retinopathy progression, possibly mediated in part by rapid glycemic improvement in susceptible individuals. Given the heterogeneous and conflicting evidence, well-designed prospective trials focused on retinal safety are needed to clarify causality and guide clinical risk stratification.

## Figures and Tables

**Table 1:** Characteristics and findings of studies evaluating the association between GLP-1RAs and non-arteritic anterior ischemic optic neuropathy (NAION).

Study	Study Design	Key Findings	Ref.
Cai et al., 2025	Retrospective cohort study, Adults with T2D taking semaglutide, dulaglutide, exenatide vs. non–GLP-1RA medications (empagliflozin, sitagliptin, glipizide)	An increased risk of NAION was observed with semaglutide and exenatide, whereas dulaglutide and non-GLP-1RA comparators (empagliflozin, sitagliptin, glipizide) showed no elevated risk.	[66]
Grauslund et al., 2024	Retrospective cohort study; semaglutide vs. none GLP-1Ras; T2DM subjects	Once-weekly semaglutide was independently associated with a more than twofold increased risk of NAION (HR 2.19, 95% CI 1.54–3.12), with a median time to onset of 22.2 months.	[57]
Hathaway et al., 2024	Retrospective cohort study, T2DM or obese patients receiving semaglutide vs. propensity-matched non-GLP-1RA controls.	Over 36 months, semaglutide was associated with a markedly higher cumulative incidence and significantly increased hazard of NAION compared with non-GLP-1RA therapies.	[56]
Simonsen et al., 2025	Retrospective cohort study; semaglutide vs. SGLT-2is, T2DM subjects	Semaglutide use was associated with a more than twofold higher risk of NAION compared with SGLT-2 inhibitors (HR 2.81, 95% CI 1.67–4.75).	[67]
Nogueira A. et al., 2026	Meta-analysis, eight retrospective cohort studies	GLP-1 RA use was associated with a modest increase in NAION risk (OR: 1.70)	[68]

NAION: non-arteritic anterior ischemic optic neuropathy; GLP-1 RA: glucagon-like peptide-1 receptor agonist; T2DM: type 2 diabetes mellitus; SGLT-2i: sodium–glucose cotransporter-2 inhibitor; HR: hazard ratio; OR: odds ratio; CI: confidence interval; Ref.: reference.

**Table 2:** Characteristics and findings of studies evaluating the association between GLP-1RAs and diabetic macular edema, macular hole, retinal detachment and vitreous hemorrhage.

Study	Study Design	Key Findings	Ref.
Diabetic Macular Edema			
Wai et al., 2024	Retrospective cohort study, NPDR patients under GLP-1 RA or SGLT-2i monotherapy	Higher risk of new-onset DME in 3,6,12 and 36 months with GLP-1 RAs vs. SGLT-2i (RR: 1.29, CI 1.21–1.38, *p* < 0.001, at 3 years)	[78]
Talebi et al., 2025	Longitudinal retrospective cohort study, GLP-1 RA users and never-users with T2DM followed for 10 years	Decreased risk of DME with GLP-1RA use (HR: 0.40, 95% CI: 0.27–0.59, *p* < 0.001)	[72]
Muayad et al., 2025	Retrospective cohort study, T2DM patients, newly initiated on GLP-1RAs, compared to propensity score-matched controls	Decreased risk of DME with GLP-1RA use (HR: 0.77, 95% CI: 0.70–0.85)	[73]
Tauqeer et al.	Retrospective cohort study, T2DM patients with NPDR on GLP-1RA therapy compared to non-users of GLP-1RA	No evidence of progression to DME in GLP-1RA users (HR = 1.06, 95% CI: 0.95–1.1.9, *p* = 0.31)	[75]
Marso et al., 2025	RCT, T2DM randomly assigned to semaglutide or placebo treatment once weekly	The semaglutide group had a significantly higher risk of retinopathy complications requiring intravitreal agents or photocoagulation (possibly including DME cases, HR: 1.76; 95% CI, 1.11 to 2.78; *p* = 0.02)	[4]
Avgerinos et al., 2019	Meta-analysis of RCTs to assess microvascular endpoints with the use of GLP-1RAs, 60 studies included	There was no significant risk of DME compared either with placebo (OR 0.84; 95% CI, 0.44–1.57) or another antidiabetic treatment (OR 1.14; 95% CI, 0.34–3.84).	[79]
Macular Hole			
Lakhani et al., 2025	Global pharmacovigilance study of optic nerve and retinal AEs with semaglutide or tirzepatide	Increased SDR for macular holes with semaglutide (ROR 20.90, 95% CI 2.65–165.01)	[77]
Retinal Detachment			
Joo et al., 2024	Retrospective cohort study, patients with DM under GLP-1RA or SGLT2i treatment	There was no statistically significant difference in the simultaneous event of vitreous hemorrhage and RD (OR 1.50, 95% CI 0.25–8.98).	[86]
Vitreous Hemorrhage			
Ramsey et al., 2025	Retrospective cohort study, T2DM adults with a recent Hb1Ac of 6.5% or higher divided into two groups based on whether they were prescribed GLP-1RAs	Lower incidence of VH in the GLP-1RA group compared with the control group of no GLP-1RA use (HR 0.74; 95% CI 0.68–0.80; *p* < 0.001)	[74]
Avgerinos et al., 2019	Meta-analysis of RCTs to assess microvascular endpoints with the use of GLP-1RAs, 60 studies included	Higher incidence of VH in patients treated with GLP-1RAs compared with those receiving placebo (OR 1.93; 95% CI 1.09–3.42)	[79]

NPDR: Non-proliferative diabetic retinopathy; GLP-1 RA: glucagon-like peptide-1 receptor agonist; SGLT-2i: sodium–glucose cotransporter-2 inhibitor; DME: Diabetic Macular Edema; T2DM: type 2 diabetes mellitus; RCT: randomized controlled trial; SDR: signal of disproportionate reporting; AEs: adverse events; RD: retinal detachment; VH: vitreous hemorrhage.

## Data Availability

The original contributions presented in this study are included in the article. Further inquiries can be directed to the corresponding author.
